# *N*-(4-Eth­oxy-2,5-di­nitro­phen­yl)acetamide

**DOI:** 10.1107/S2414314620011219

**Published:** 2020-08-28

**Authors:** Sannihith N. Uppu, Ogad A. Agu, Curtistine J. Deere, Frank R. Fronczek

**Affiliations:** a Department of Biological Engineering, Louisiana State University, Baton Rouge, LA, 70803, USA; b Department of Environmental Toxicology, Southern University and A&M College, Baton Rouge, LA, 70813, USA; c Department of Chemistry, Louisiana State University, Baton Rouge, LA 70803, USA; University of Aberdeen, Scotland

**Keywords:** crystal structure, nitrated phenacetin, hydrogen bonding

## Abstract

In the title compound, C_10_H_11_N_3_O_6_, the torsion angles about the bonds to the benzene ring are less than 4°, except for the nitro groups, which are twisted out of the ring plane by 25.27 (3) and 43.63 (2)°. The N—H group forms a bifurcated hydrogen bond, with an intra­molecular component to a nitro group O atom and an inter­molecular component to the other nitro group, thereby forming chains propagating in the [010] direction. Several weak C—H⋯O inter­actions are also present.

## Abstract 2

In the title compound, the nitro groups are rotated significantly out of the plane of the benzene ring and the amine group forms a bifurcated N—H⋯(O,O) hydrogen bond with intra­molecular and inter­molecular components.

## Graphical abstract

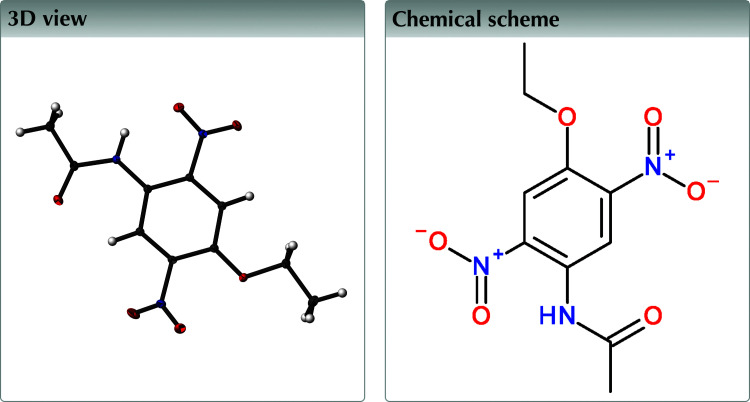

## Structure description

The analgesic use of 4-acetamido­phenetole (4-AcP) predates the First World War. 4-AcP was likely the first synthetic chemical to go on the market as a fever reducer, but was withdrawn from global markets three decades ago due to its carcinogenic and kidney-damaging properties (Zeman, 1963 ▸; Carrociampi, 1978 ▸; Leistenschneider *et al.*, 1983 ▸; Holmäng *et al.*, 2013 ▸). However, in view of 4-AcP’s physical appearance and textural similarities to cocaine, in recent years, there have been several instances of 4-AcP being used as an adulterant or cutting agent (Broséus *et al.*, 2016 ▸). Thus, phenacetin is still in use, however, now in the form of an illicit drug. We believe that 4-AcP, like its putative major metabolite, 4-acetamido­phenol (4-AP) (Hinson, 1983 ▸; Lakshmi *et al.*, 2000 ▸; Liu *et al.*, 2019 ▸), undergoes oxidative transformation by cellular oxidants such as hypochlorite/hypo­chlorous acid and per­oxy­nitrite/per­oxy­nitrous acid and forms chlorinated and nitrated products. Towards understanding this and to shed light on mol­ecular targets, we have synthesized the title compound 2,5-di­nitro-4-AcP, C_10_H_11_N_3_O_6_, and we now report its structure. The results of the present study, together with the recent understanding of the mechanisms of action of 4-acetamido­phenol (4-AP), which proceeds through hydrolysis and subsequent formation of arachidonic acid conjugates and their binding cannabinoid receptors, may be useful in providing insights into mol­ecular targets for 4-AcP and its metabolites.

**Figure 1:**
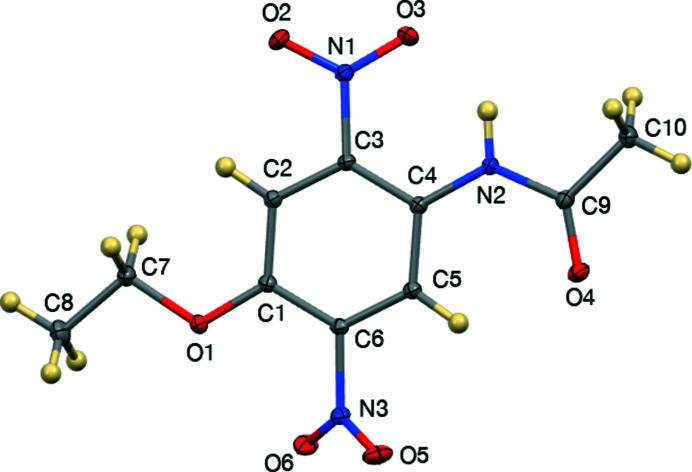
The title mol­ecule showing 50% displacement ellipsoids.

The eth­oxy group is nearly coplanar with the phenyl ring, having a C2—C1—O1—C7 torsion angle of 1.43 (8)° and C1—O1—C7—C8(Me) torsion angle of 174.56 (5)°, as shown in Fig. 1 ▸. The acetamido group is also nearly coplanar with the phenyl ring, having a C5—C4—N2—C9 torsion angle of 3.18 (9)°. The N1/O2/O3 nitro group adjacent to the acetamido substituent is twisted out of the phenyl plane by 25.27 (3)°, and the N3/O5/O6 group adjacent to the eth­oxy group forms a dihedral angle of 43.63 (2)° with respect to the C1–C6 ring.

**Figure 2:**
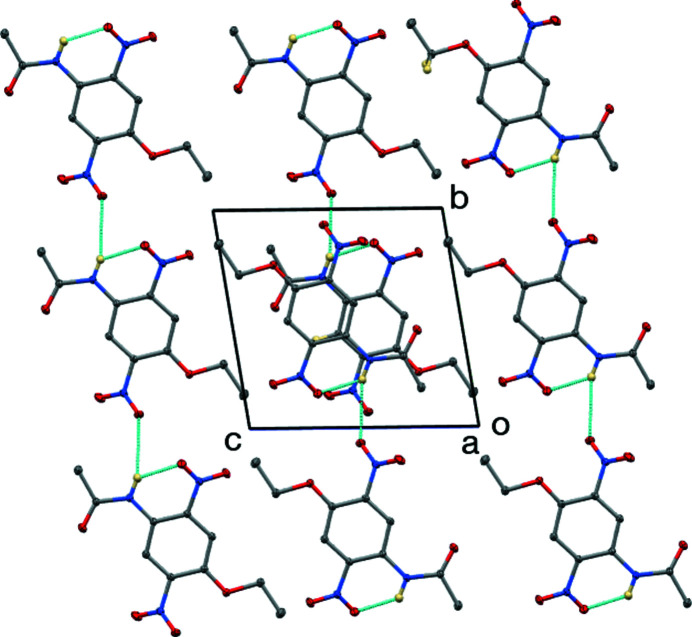
The unit cell viewed down [100], showing hydrogen bonds as blue lines. C—H hydrogen atoms are not shown.

The N2—H2*N* group forms a bifurcated hydrogen bond (Table 1 ▸), with an intra­molecular component to the adjacent nitro group [N2⋯O3 = 2.6875 (6) Å] and a longer inter­molecular component to the other nitro group [N2⋯O6^i^ = 3.4308 (6) Å; symmetry code: (i) *x*, *y* + 1, *z*], forming chains propagating in the [010] direction, as shown in Fig. 2 ▸. Several C—H⋯O inter­actions are also present (Table 1 ▸), which together with the N—H⋯O hydrogen bond lead to (100) sheets.

## Synthesis and crystallization

2,5-Di­nitro-4-AcP was synthesized by nitration of 4-AcP using nitric acid–sulfuric acid mixtures (0–5°C) and subsequent purification by column chromatography on alumina or silica gel as described by Russell *et al.* (1990 ▸). Yellow needles were grown by slow evaporation from methanol solution.

## Refinement

Crystal data, data collection and structure refinement details are summarized in Table 2 ▸.

## Supplementary Material

Crystal structure: contains datablock(s) I. DOI: 10.1107/S2414314620011219/hb4359sup1.cif

Structure factors: contains datablock(s) I. DOI: 10.1107/S2414314620011219/hb4359Isup2.hkl

Supporting information file. DOI: 10.1107/S2414314620011219/hb4359Isup3.cml

CCDC reference: 2023527

Additional supporting information:  crystallographic information; 3D view; checkCIF report

## Figures and Tables

**Table 1:** Hydrogen-bond geometry (Å, °)

*D*—H⋯*A*	*D*—H	H⋯*A*	*D*⋯*A*	*D*—H⋯*A*
N2—H2*N*⋯O3	0.900 (10)	2.015 (10)	2.6875 (6)	130.5 (8)
N2—H2*N*⋯O6^i^	0.900 (10)	2.618 (10)	3.4308 (6)	150.5 (8)
C5—H5*A*⋯O4	0.95	2.20	2.8386 (7)	123
C8—H8*A*⋯O2^ii^	0.98	2.63	3.3768 (9)	133
C10—H10*A*⋯O2^iii^	0.98	2.37	3.3367 (7)	171
C10—H10*B*⋯O5^i^	0.98	2.65	3.5677 (8)	155

Symmetry codes: (i) 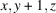 ; (ii) 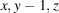 ; (iii) 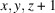 .

**Table 2:** Experimental details

Crystal data	
Chemical formula	C_10_H_11_N_3_O_6_
*M*_r_	269.22
Crystal system, space group	Triclinic, *P* 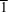
Temperature (K)	90
*a*, *b*, *c* (Å)	6.7463 (3), 9.0360 (4), 9.3954 (4)
α, β, γ (°)	81.005 (2), 85.099 (2), 88.700 (2)
*V* (Å^3^)	563.60 (4)
*Z*	2
Radiation type	Mo *K*α
μ (mm^−1^)	0.13
Crystal size (mm)	0.30 × 0.10 × 0.09

Data collection	
Diffractometer	Bruker Kappa APEXII DUO CCD
Absorption correction	Multi-scan (*SADABS*; Krause *et al.*, 2015 ▸)
*T*_min_, *T*_max_	0.920, 0.988
No. of measured, independent and observed [*I* > 2σ(*I*)] reflections	29518, 7110, 5824
*R*_int_	0.043
(sin θ/λ)_max_ (Å^−1^)	0.911

Refinement	
*R*[*F* ^2^ > 2σ(*F* ^2^)], *wR*(*F* ^2^), *S*	0.038, 0.111, 1.04
No. of reflections	7110
No. of parameters	177
H-atom treatment	H atoms treated by a mixture of independent and constrained refinement
Δρ_max_, Δρ_min_ (e Å^−3^)	0.76, −0.28

Computer programs: *APEX3* and *SAINT* (Bruker, 2016 ▸), *SHELXS97* (Sheldrick, 2008 ▸), *SHELXL2017/1* (Sheldrick, 2015 ▸), *Mercury* (Macrae *et al.*, 2020 ▸) and *publCIF* (Westrip, 2010 ▸).

## full crystallographic data

### Crystal data

**Table:**

C_10_H_11_N_3_O_6_	*Z* = 2
*M**_r_* = 269.22	*F*(000) = 280
Triclinic, *P*1	*D*_x_ = 1.586 Mg m^−^^3^
*a* = 6.7463 (3) Å	Mo *K*α radiation, λ = 0.71073 Å
*b* = 9.0360 (4) Å	Cell parameters from 9965 reflections
*c* = 9.3954 (4) Å	θ = 3.0–40.2°
α = 81.005 (2)°	µ = 0.13 mm^−^^1^
β = 85.099 (2)°	*T* = 90 K
γ = 88.700 (2)°	Needle, yellow
*V* = 563.60 (4) Å^3^	0.30 × 0.10 × 0.09 mm

### Data collection

**Table:**

Bruker Kappa APEXII DUO CCD diffractometer	7110 independent reflections
Radiation source: fine-focus sealed tube	5824 reflections with *I* > 2σ(*I*)
TRIUMPH curved graphite monochromator	*R*_int_ = 0.043
φ and ω scans	θ_max_ = 40.4°, θ_min_ = 2.2°
Absorption correction: multi-scan (SADABS; Krause *et al.*, 2015)	*h* = −11→12
*T*_min_ = 0.920, *T*_max_ = 0.988	*k* = −15→16
29518 measured reflections	*l* = −17→15

### Refinement

**Table:**

Refinement on *F*^2^	Primary atom site location: structure-invariant direct methods
Least-squares matrix: full	Hydrogen site location: mixed
*R*[*F*^2^ > 2σ(*F*^2^)] = 0.038	H atoms treated by a mixture of independent and constrained refinement
*wR*(*F*^2^) = 0.111	*w* = 1/[σ^2^(*F*_o_^2^) + (0.0597*P*)^2^ + 0.0585*P*] where *P* = (*F*_o_^2^ + 2*F*_c_^2^)/3
*S* = 1.04	(Δ/σ)_max_ = 0.001
7110 reflections	Δρ_max_ = 0.76 e Å^−^^3^
177 parameters	Δρ_min_ = −0.28 e Å^−^^3^
0 restraints	

### Special details

**Table:**

Geometry. All esds (except the esd in the dihedral angle between two l.s. planes) are estimated using the full covariance matrix. The cell esds are taken into account individually in the estimation of esds in distances, angles and torsion angles; correlations between esds in cell parameters are only used when they are defined by crystal symmetry. An approximate (isotropic) treatment of cell esds is used for estimating esds involving l.s. planes.
Refinement. All H atoms were located in difference maps and those on C were thereafter treated as riding in geometrically idealized positions with C—H distances 0.95 Å for phenyl, 0.99 Å for CH_2_ and 0.98 Å for methyl. Coordinates of the N—H hydrogen atom were refined. *U*_iso_(H) values were assigned as 1.2*U*_eq_ for the attached C or N atom (1.5 for methyl).

### Fractional atomic coordinates and isotropic or equivalent isotropic displacement parameters (Å^2^)

**Table:**

	*x*	*y*	*z*	*U*_iso_*/*U*_eq_	
O1	0.69178 (7)	0.25306 (4)	0.22213 (4)	0.01208 (7)	
O2	0.80562 (9)	0.79863 (5)	0.13502 (5)	0.02166 (10)	
O3	0.94649 (7)	0.84282 (5)	0.32212 (5)	0.01549 (8)	
O4	0.74286 (8)	0.55569 (5)	0.80156 (5)	0.01579 (8)	
O5	0.54980 (7)	0.15057 (5)	0.63040 (5)	0.01655 (9)	
O6	0.76371 (8)	0.07153 (5)	0.47211 (5)	0.01675 (9)	
N1	0.84988 (7)	0.76273 (5)	0.25917 (5)	0.01119 (8)	
N2	0.78930 (7)	0.69046 (5)	0.57358 (5)	0.01020 (7)	
H2N	0.8235 (15)	0.7816 (11)	0.5253 (10)	0.012*	
N3	0.66942 (7)	0.17087 (5)	0.52301 (5)	0.01052 (7)	
C1	0.72020 (8)	0.36104 (5)	0.30173 (5)	0.00923 (8)	
C2	0.76108 (8)	0.50980 (6)	0.24458 (5)	0.00986 (8)	
H2A	0.768693	0.540555	0.142853	0.012*	
C3	0.79085 (8)	0.61374 (5)	0.33497 (5)	0.00893 (8)	
C4	0.77060 (7)	0.58036 (5)	0.48682 (5)	0.00857 (8)	
C5	0.72406 (7)	0.43112 (5)	0.54384 (5)	0.00912 (8)	
H5A	0.705143	0.401790	0.645627	0.011*	
C6	0.70546 (7)	0.32621 (5)	0.45315 (5)	0.00893 (8)	
C7	0.71005 (9)	0.29652 (6)	0.06650 (6)	0.01309 (9)	
H7A	0.840288	0.344280	0.034497	0.016*	
H7B	0.603451	0.368885	0.036447	0.016*	
C8	0.69258 (11)	0.15636 (7)	0.00051 (7)	0.01860 (11)	
H8A	0.799779	0.086200	0.030043	0.028*	
H8B	0.702903	0.181958	−0.105121	0.028*	
H8C	0.563670	0.109681	0.033650	0.028*	
C9	0.77619 (8)	0.67490 (6)	0.72252 (5)	0.01021 (8)	
C10	0.80679 (9)	0.81975 (6)	0.77720 (6)	0.01351 (9)	
H10A	0.805013	0.800989	0.882891	0.020*	
H10B	0.699950	0.890571	0.748418	0.020*	
H10C	0.935357	0.862121	0.735925	0.020*	

### Atomic displacement parameters (Å^2^)

**Table:**

	*U*^11^	*U*^22^	*U*^33^	*U*^12^	*U*^13^	*U*^23^
O1	0.01876 (18)	0.00903 (15)	0.00891 (14)	−0.00178 (12)	−0.00191 (12)	−0.00211 (11)
O2	0.0418 (3)	0.01400 (18)	0.00889 (16)	−0.00649 (18)	−0.00647 (17)	0.00250 (13)
O3	0.0220 (2)	0.01191 (16)	0.01286 (16)	−0.00698 (14)	−0.00066 (14)	−0.00239 (13)
O4	0.0251 (2)	0.01173 (16)	0.00957 (15)	−0.00162 (14)	−0.00047 (14)	0.00104 (12)
O5	0.01628 (19)	0.01397 (17)	0.01650 (18)	−0.00063 (14)	0.00464 (14)	0.00348 (14)
O6	0.0270 (2)	0.00892 (16)	0.01395 (17)	0.00363 (14)	0.00026 (15)	−0.00213 (13)
N1	0.01583 (19)	0.00887 (16)	0.00837 (15)	−0.00144 (13)	0.00098 (13)	−0.00063 (12)
N2	0.01441 (18)	0.00899 (16)	0.00720 (15)	−0.00176 (13)	−0.00061 (13)	−0.00115 (12)
N3	0.01234 (18)	0.00852 (16)	0.01048 (16)	−0.00062 (13)	−0.00219 (13)	−0.00006 (12)
C1	0.01055 (18)	0.00821 (17)	0.00896 (17)	−0.00029 (13)	−0.00101 (13)	−0.00125 (13)
C2	0.01238 (19)	0.00864 (17)	0.00846 (17)	−0.00046 (14)	−0.00077 (14)	−0.00106 (13)
C3	0.01064 (18)	0.00772 (16)	0.00810 (16)	−0.00083 (13)	−0.00041 (13)	−0.00031 (13)
C4	0.00907 (17)	0.00864 (17)	0.00797 (16)	−0.00039 (13)	−0.00065 (13)	−0.00115 (13)
C5	0.01008 (18)	0.00848 (17)	0.00854 (17)	−0.00012 (13)	−0.00084 (13)	−0.00044 (13)
C6	0.00947 (18)	0.00756 (17)	0.00939 (17)	−0.00010 (13)	−0.00098 (13)	−0.00012 (13)
C7	0.0193 (2)	0.01123 (19)	0.00900 (18)	−0.00058 (16)	−0.00162 (16)	−0.00213 (14)
C8	0.0270 (3)	0.0157 (2)	0.0145 (2)	−0.0029 (2)	−0.0004 (2)	−0.00697 (18)
C9	0.01131 (19)	0.01111 (18)	0.00811 (17)	0.00027 (14)	−0.00081 (13)	−0.00121 (14)
C10	0.0185 (2)	0.0127 (2)	0.00990 (18)	−0.00142 (16)	−0.00154 (16)	−0.00304 (15)

### Geometric parameters (Å, º)

**Table:**

O1—C1	1.3451 (6)	C2—H2A	0.9500
O1—C7	1.4489 (7)	C3—C4	1.4068 (7)
O2—N1	1.2214 (6)	C4—C5	1.4031 (7)
O3—N1	1.2327 (6)	C5—C6	1.3845 (7)
O4—C9	1.2225 (7)	C5—H5A	0.9500
O5—N3	1.2299 (6)	C7—C8	1.5058 (8)
O6—N3	1.2228 (6)	C7—H7A	0.9900
N1—C3	1.4677 (7)	C7—H7B	0.9900
N2—C9	1.3798 (7)	C8—H8A	0.9800
N2—C4	1.3953 (6)	C8—H8B	0.9800
N2—H2N	0.900 (10)	C8—H8C	0.9800
N3—C6	1.4698 (7)	C9—C10	1.5035 (8)
C1—C2	1.3918 (7)	C10—H10A	0.9800
C1—C6	1.4036 (7)	C10—H10B	0.9800
C2—C3	1.3895 (7)	C10—H10C	0.9800
			
C1—O1—C7	116.70 (4)	C5—C6—C1	123.59 (4)
O2—N1—O3	123.54 (5)	C5—C6—N3	116.61 (4)
O2—N1—C3	117.91 (5)	C1—C6—N3	119.79 (4)
O3—N1—C3	118.51 (4)	O1—C7—C8	107.33 (4)
C9—N2—C4	128.40 (4)	O1—C7—H7A	110.2
C9—N2—H2N	116.4 (6)	C8—C7—H7A	110.2
C4—N2—H2N	115.0 (6)	O1—C7—H7B	110.2
O6—N3—O5	124.77 (5)	C8—C7—H7B	110.2
O6—N3—C6	117.66 (4)	H7A—C7—H7B	108.5
O5—N3—C6	117.55 (4)	C7—C8—H8A	109.5
O1—C1—C2	124.44 (5)	C7—C8—H8B	109.5
O1—C1—C6	119.56 (4)	H8A—C8—H8B	109.5
C2—C1—C6	115.99 (4)	C7—C8—H8C	109.5
C3—C2—C1	120.59 (4)	H8A—C8—H8C	109.5
C3—C2—H2A	119.7	H8B—C8—H8C	109.5
C1—C2—H2A	119.7	O4—C9—N2	123.41 (5)
C2—C3—C4	123.56 (4)	O4—C9—C10	123.63 (5)
C2—C3—N1	114.48 (4)	N2—C9—C10	112.96 (4)
C4—C3—N1	121.95 (4)	C9—C10—H10A	109.5
N2—C4—C5	122.78 (4)	C9—C10—H10B	109.5
N2—C4—C3	121.68 (4)	H10A—C10—H10B	109.5
C5—C4—C3	115.50 (4)	C9—C10—H10C	109.5
C6—C5—C4	120.61 (4)	H10A—C10—H10C	109.5
C6—C5—H5A	119.7	H10B—C10—H10C	109.5
C4—C5—H5A	119.7		
			
C7—O1—C1—C2	1.43 (8)	N2—C4—C5—C6	179.44 (5)
C7—O1—C1—C6	−179.73 (5)	C3—C4—C5—C6	1.54 (7)
O1—C1—C2—C3	−179.20 (5)	C4—C5—C6—C1	−3.56 (8)
C6—C1—C2—C3	1.92 (8)	C4—C5—C6—N3	176.11 (5)
C1—C2—C3—C4	−3.97 (8)	O1—C1—C6—C5	−177.20 (5)
C1—C2—C3—N1	174.85 (5)	C2—C1—C6—C5	1.74 (8)
O2—N1—C3—C2	23.69 (7)	O1—C1—C6—N3	3.14 (7)
O3—N1—C3—C2	−154.05 (5)	C2—C1—C6—N3	−177.92 (5)
O2—N1—C3—C4	−157.47 (6)	O6—N3—C6—C5	−136.01 (5)
O3—N1—C3—C4	24.79 (8)	O5—N3—C6—C5	42.40 (7)
C9—N2—C4—C5	3.18 (9)	O6—N3—C6—C1	43.67 (7)
C9—N2—C4—C3	−179.04 (5)	O5—N3—C6—C1	−137.92 (5)
C2—C3—C4—N2	−175.80 (5)	C1—O1—C7—C8	174.56 (5)
N1—C3—C4—N2	5.47 (8)	C4—N2—C9—O4	−0.69 (9)
C2—C3—C4—C5	2.13 (8)	C4—N2—C9—C10	179.41 (5)
N1—C3—C4—C5	−176.60 (5)		

### Hydrogen-bond geometry (Å, º)

**Table:**

*D*—H···*A*	*D*—H	H···*A*	*D*···*A*	*D*—H···*A*
N2—H2*N*···O3	0.900 (10)	2.015 (10)	2.6875 (6)	130.5 (8)
N2—H2*N*···O6^i^	0.900 (10)	2.618 (10)	3.4308 (6)	150.5 (8)
C5—H5*A*···O4	0.95	2.20	2.8386 (7)	123
C8—H8*A*···O2^ii^	0.98	2.63	3.3768 (9)	133
C10—H10*A*···O2^iii^	0.98	2.37	3.3367 (7)	171
C10—H10*B*···O5^i^	0.98	2.65	3.5677 (8)	155

Symmetry codes: (i) *x*, *y*+1, *z*; (ii) *x*, *y*−1, *z*; (iii) *x*, *y*, *z*+1.

## References

[bb1] Broséus, J., Gentile, N. & Esseiva, P. (2016). *Forensic Sci. Int.* **262**, 73–83. 10.1016/j.forsciint.2016.02.033 26974713

[bb2] Bruker (2016). *APEX2* and *SAINT*. Bruker AXS Inc., Madison, Wisconsin, USA.

[bb3] Carrociampi, G. (1978). *Toxicology*, **10**, 311–339.

[bb4] Hinson, J. A. (1983). *Environ. Health Perspect.* **49**, 71–79. 10.1289/ehp.834971 PMC1569121 6339229

[bb5] Holmäng, S., Holmberg, E. & Johansson, S. L. (2013). *Scand. J. Urol.* **47**, 491–496. 10.3109/21681805.2013.795188 23634644

[bb6] Krause, L., Herbst-Irmer, R., Sheldrick, G. M. & Stalke, D. (2015). *J. Appl. Cryst.* **48**, 3–10. 10.1107/S1600576714022985 PMC4453166 26089746

[bb7] Lakshmi, V. M., Hsu, F. F., Davis, B. B. & Zenser, T. V. (2000). *Chem. Res. Toxicol.* **13**, 891–899. 10.1021/tx000115g 10995262

[bb8] Leistenschneider, W., Nagel, R. & Steffens, J. (1983). *Aktuel. Urol.* **14**, 15–20.

[bb9] Liu, Y. J., Liu, H. S., Hu, C. Y. & Lo, S. L. (2019). *Water Res.* **155**, 56–65. 10.1016/j.watres.2019.01.061 30831424

[bb10] Macrae, C. F., Sovago, I., Cottrell, S. J., Galek, P. T. A., McCabe, P., Pidcock, E., Platings, M., Shields, G. P., Stevens, J. S., Towler, M. & Wood, P. A. (2020). *J. Appl. Cryst.* **53**, 226–235. 10.1107/S1600576719014092 PMC6998782 32047413

[bb11] Russell, R. A., Switzer, R. W., Longmore, R. W., Dutton, B. H. & Harland, L. (1990). *J. Chem. Educ.* **67**, 168–169.

[bb12] Sheldrick, G. M. (2008). *Acta Cryst.* A**64**, 112–122. 10.1107/S0108767307043930 18156677

[bb13] Sheldrick, G. M. (2015). *Acta Cryst.* C**71**, 3–8.

[bb14] Westrip, S. P. (2010). *J. Appl. Cryst.* **43**, 920–925.

[bb15] Zeman, F. D. (1963). *J. Chronic Dis.* **16**, 1085–1098. 10.1016/0021-9681(63)90043-2 14068921

